# The Mediating Effect of Stress between Extracurricular Activities and Suicidal Ideation in Chinese College Students

**DOI:** 10.3390/ijerph20043105

**Published:** 2023-02-10

**Authors:** Tao Wang, Zhiying Yao, Qianqian Liu, Jingjing Zhao, Xinting Wang, Josephine Pui-Hing Wong, Mandana Vahabi, Cunxian Jia

**Affiliations:** 1 Department of Epidemiology, School of Public Health, Cheeloo College of Medicine, Shandong University Center for Suicide Prevention Research, Shandong University, Jinan 250012, China; 2 Student Counseling Center, Shandong University, Jinan 250012, China; 3 School of Public Health, Cheeloo College of Medicine, Shandong University, Jinan 250012, China; 4 Daphne Cockwell School of Nursing, Toronto Metropolitan University, Toronto, ON M5B 1Z5, Canada

**Keywords:** suicidal ideation, stress, extracurricular activities, college students

## Abstract

Objective: The objective of this study was to explore the relationship between extracurricular activities, stress, and suicidal ideation and to examine the mediating effect of stress between extracurricular activities and suicidal ideation in Chinese college students. Method: A total of 6446 college students were surveyed with a web-based online data collection system using the self-made demographic questionnaire, Suicidal Behaviors Questionnaire—Revised (SBQ-R), and the 21-Item Depression Anxiety Stress Scales (DASS-21). SPSS 24.0 was used for descriptive statistics and correlation analysis, and the bootstrap method in the process procedure for SPSS Version 3.4.1 was used to construct the mediating effect model. Results: Gender, school grades, living area, and family income status were influencing factors for suicidal ideation, stress, and extracurricular activities. Extracurricular activities were negatively correlated with stress (*r* = −0.083, *p* < 0.001) and suicidal ideation (*r* = −0.039, *p* < 0.01). Extracurricular activities had no direct predictive effect on college students’ suicidal ideation (*c* = −0.198, CI: −0.418, 0.023), while stress had a mediating effect between extracurricular activities and suicidal ideation; the indirect mediating effect was 0.159. Conclusions: Extracurricular activities indirectly predict college students’ suicidal ideation through stress. A variety of extracurricular activities can decrease the stress and suicidal ideation of college students and benefit their mental health.

## 1. Introduction

Suicide is a serious global public health issue. According to the report of the World Health Organization (WHO) [1], *Suicide Worldwide in 2019: Global Health Estimates,* more than 700,000 persons die by suicide every year globally. Under the guidance of suicide intervention and preventive measures by WHO and many countries, the global suicide mortality rate has decreased by one-third [2]. With the implementation of relevant laws and policies regarding mental health, the overall suicide rate in China has also been declining in the past three decades, from 20.9/100,000 in 1990 to 7.2/100,000 in 2017 [3]. However, China’s total suicide-related deaths still account for 15% of the global rates, which is considered to be significant [4].

Suicide has an immeasurable negative impact on the person’s family and society. According to previous research, it is estimated that each suicide death will affect at least 135 people, including family, friends, and colleagues [5]. Given the severe consequences of suicide deaths, it is important to identify risk factors that can be acted on to reduce or prevent suicide. Suicide includes suicidal ideation, suicide attempts, and suicide plans. Suicidal ideation is defined as thoughts of ending one’s life and is considered a strong predictor of suicide [6,7,8]. College students with suicidal ideation have a high risk of suicide [9]. 

A few studies have demonstrated that suicide is the leading cause of mortality among 10- to 24-year-olds [10,11]. One study postulated that the suicide rate among Chinese college students is between 1 in 100,000 and 3 in 100,000 [12]. College students are a unique subgroup within this population because they are faced with different physical, psychological, social, and intellectual demands. In addition to developmental, physical, and psychological changes, college students are expected to take on evolving social roles and develop new relationships; for example, demonstrating the ability to lead a pro-active, semi-independent life after leaving parents, engaging in romantic love, building friendships with new classmates, etc. As a result of the significant social demands and changes in lifestyles and learning styles, college students are more likely to experience mental problems, including suicidal ideation. Multiple studies have shown a high prevalence of suicidal ideation among college students [13,14,15,16]. It is well-known that suicidal ideation is related to various factors, including mental disorders, physical illnesses, and biological, psychosocial, and environmental factors [17,18,19,20]. Although many risk factors and protective factors have been identified, suicidal ideation is still challenging to prevent [21]. Therefore, the effective intervention and regular monitoring of suicidal ideation among college students are necessary to reduce the long-term risk of suicide.

Mental health problems are common among college students [22,23]. Chronic stress plays a crucial role in developing psychiatric diseases such as anxiety and depression [24]. Therefore, reducing the stress experienced by college students can also reduce other psychological symptoms. Many studies have revealed that the prevalence of stress among college students is high. A survey of students at Krakow University in Poland by Anna Średniawa et al. [25] showed that 43% had moderate or high stress levels. Studies on Chinese college students have also shown that the prevalence of stress is 20.2~36.9% [26,27]. Furthermore, stress is not only a negative predictor of recent suicidal ideation in college students [28,29,30] but is also an indicator of chronic suicidal ideation [31].

The sources of stress among college students are multifaceted and include demands related to academic achievements, interpersonal relationships, concerns about future careers, etc. Methods for alleviating stress are mainly physiological and psychological. College students’ physical stress-relief methods include physical activity, such as sports activities and dancing [32,33], and daily leisure activities, such as cooking and hobbies [34]. The physical methods of relieving stress are easy to implement and diverse. Psychologically, positive interpersonal relationships with parents, teachers, and peers—that is, social–emotional support—are an important way to reduce stress [35]. Some studies [36,37] have reported that participation in extracurricular activities helps to reduce suicide-related outcomes, but other studies have not been able to confirm this finding [38,39]. Considering previous studies (e.g., Schuch et al. [40]; Stubbs et al. [41]), it can be hypothesized that stress, as a negative dimension of mental health, may mediate this relationship.

The purpose of this study was to analyze the possible mediating effect of stress between extracurricular activities and suicidal ideation and to provide a reference for strategies to reduce suicidal ideation among college students.

## 2. Participants and Methods

### 2.1. Study Overview and Participants

A total of seven universities in Shandong, Shaanxi, and Jilin Provinces in China were selected using convenience sampling. Two to four colleges were selected from each university, and one to two classes were selected for each grade in each college, with all students in the class participating in this survey. From September 2020 to January 2021, a cross-sectional survey of college students’ mental health was conducted through a web-based online data collection system. Informed consent was obtained from all participants. A consistent protocol was used across all seven universities. A total of 6446 valid questionnaires were collected. Of the participants, 2435 were male, accounting for 37.78%, and 4011 were female, accounting for 62.22%. The average age was 19.49 (SD:1.28) years old. 

### 2.2. Data Collection

#### 2.2.1. Demographic Questionnaire

A questionnaire was used to collect the college students’ demographic data, including gender (male/female), age, school grades (1, 2, 3, and above), subject (medical/non-medical), living area (urban/rural), one-child status (yes/no), family income status (poor, fair, good), height, weight, etc. The family income status was self-evaluated and not classified by actual income. 

#### 2.2.2. Suicidal Ideation

The Suicidal Behaviors Questionnaire—Revised (SBQ-R) scale by Osman et al. [42] was used to investigate whether there was suicidal ideation over a lifetime. This scale contains four items on suicidal behaviors and ideations over the lifetime and over the past 12 months, suicide-related communication, and self-reported likelihood of any future suicidal behavior. Suicidal ideation was evaluated by the item: “How often have you thought about killing yourself in the past year?”. This item had 5 options, 1 = Never, 2 = Rarely (1 time), 3 = Sometimes (2 times), 4 = Often (3–4 times), and 5 = Very Often (5 or more times). If the participant answered 2–5, we categorized them as having suicidal ideation. The internal reliability of the scale in this study was Cronbach’s α=0.774.

#### 2.2.3. Stress

The Chinese Version of the 21-Item Depression Anxiety Stress Scales (DASS-21) [43] was used to investigate the stress levels of college students. The questionnaire has 21 items and three dimensions—depression, anxiety, and stress—with each dimension containing 7 items. The scale has four scores ranging from “0 = not conforming” to “3 = always conforming”. In calculating the total score, the score for each item is multiplied by 2 and then added together, so the total score for stress is 0–42, with a higher score indicating more severe symptoms. Scores of 0–14, 15–25, and 26–42 represented no, moderate, and severe and above stress symptoms, respectively. The internal consistency evaluation of the Chinese version of DASS-21 by Gong Xu et al. [44] showed that the total Cronbach’s α was 0.89. The internal reliability of the scale in this study was Cronbach’s α = 0.940.

#### 2.2.4. Extracurricular Activities

Participation in extracurricular activities was evaluated by the question: ‘Have you participated in extracurricular activities during your time at university?’ The answer was ‘yes’ or ‘no’. If the participant answered ‘yes’, we categorized them as participating in extracurricular activities.

### 2.3. Quality Control

In this study, quality control was divided into two parts. Firstly, the web-based online data collection system did not accept a lack of response, and students could not skip to the next question without providing an answer. Secondly, the questionnaire data completed by the college students were cross-checked by two quality control personnel through the administrative background of the online survey system to reduce the unqualified rate of the questionnaire.

### 2.4. Statistical Analyses

The chi-square test compared the differences in the qualitative data. The *t*-test and analysis of variance were used to analyze the differences between groups of quantitative data. Spearman rank correlation was used to analyze the correlation between suicidal ideation, extracurricular activities, and stress. The bootstrap method in the process procedure of SPSS Version 3.4.1 (repeated 5000 times) was used to construct the mediating effect model. We applied a probability level of 0.05 for all statistical tests. All statistical analyses were performed using SPSS 24.0 software (IBM SPSS Statistics for Windows, Version 24.0. IBM, Armonk, NY, USA).

## 3. Results

### 3.1. Demographic Analyses

Of the overall sample, 28.82% (1858/6446) reported suicidal ideation. The participation rate in extracurricular activities was 93.39% (6020/6446), and 83.23% (5365/6446) reported no stress, 14.02% (904/6446) reported mild or moderate stress, and 2.75% (177/6446) reported stress levels of severe and above. We found that suicidal ideation and stress were higher for females, second grades, non-medical, and fair family income status participants, but lower for those engaged in extracurricular activities. Suicidal ideation was higher for rural and non-one-child status participants than for urban and one-child status participants. College students of normal weight had higher stress scores and higher participation rates in extracurricular activities (see Table 1).

### 3.2. Correlation Analysis of Suicidal Ideation, Extracurricular Activities, and Stress

Table 2 presents the correlations between suicidal ideation, extracurricular activities, and stress. Participation in extracurricular activities was negatively correlated with stress (r = −0.083, *p* < 0.001) and suicidal ideation (r = −0.039, *p* < 0.01). Higher levels of stress were positively associated with suicidal ideation (r = 0.240, *p* < 0.001). 

### 3.3. The Mediating Effect of Stress between Extracurricular Activities and Suicidal Ideation

**Figure 1:**
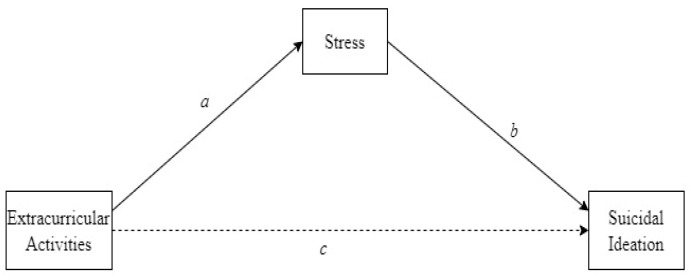
Covariates in mediation model of extracurricular activities, stress, and suicidal ideation.

Figure 1 presents the path coefficients (a, b, c) for suicidal ideation concerning extracurricular activities mediated by stress. The mediating effect was statistically significant (*R*^2^ = 0.0289, F = 31.94, *p* < 0.001). As shown in Table 3, the path coefficients of a and b were significant when predicting suicidal ideation based on extracurricular activities. With stress as the mediator, the mediation effect of stress (a∗b) was −0.159 (95% CI = −0.223, −0.114). This model showed that stress mediates the relationship between extracurricular activities and suicidal ideation; that is, participation in extracurricular activities decreases stress levels and, thus, suicidal ideation. 

## 4. Discussion

The main findings of this study were as follows. The self-report rate of suicidal ideation among 6446 college students was 28.82%, and the rates of stress and extracurricular activities were 16.77% and 93.39%, respectively. The study results showed that suicidal ideation, extracurricular activities, and stress were related to gender, school grades, discipline, family economic status, and drinking. Extracurricular activities were negatively correlated with suicidal ideation and stress, and stress was positively correlated with suicidal ideation. After controlling the covariates, stress was a full mediator between extracurricular activities and suicidal ideation. Participating in extracurricular activities can reduce the stress of college students, thereby reducing suicidal ideation.

A previous study found a causal relationship between suicidal ideation and suicide attempts and increased suicide death. Suicidal ideation is more likely to progress to suicide death [45]. This study found that the reported rate of suicidal ideation among college students was 28.82%, similar to Horgan A et al. and Eskin M et al. [14,46], but higher than other studies [16,47,48,49]. The reporting rate of suicidal ideation may be different because of the economy, culture, customs, and education system. In education, the stigma of mental illness at the school level can make students reluctant to report their suicidal ideation [50]. In terms of culture and customs, Chinese society upholds positive cultural values, such as an optimistic attitude, a belief in overcoming setbacks, etc. Suicidal ideation is usually negatively associated with immoral dimensions (i.e., harm, betrayal, and dirtiness) [51], resulting in college students feeling ashamed to report suicidal ideation.

In addition to developing academic knowledge, college students can participate in various extracurricular activities such as clubs, sports, and social practices. The availability of diverse extracurricular activities that meet the needs of students can support students to achieve balance, promoting their physical and psychosocial health [52]. More importantly, participating in extracurricular activities can enhance interpersonal communication and strengthen peer relationships, which have been shown to positively affect the mental health of college students. In this study, the participation rate in extracurricular activities was 93.39%, which was higher than in the results of Almalki SA et al. [53]. However, close to 7% of students did not participate in extracurricular activities. Since participation in extracurricular activities mediates stress, increasing uptake is important. There are numerous potential barriers to participation in extracurricular activities among college students. First, living away from home and having limited parental guidance requires college students to develop their own self-management and time management, which influence their ability to participate in extracurricular activities. Secondly, although the number of extracurricular activities is large, the types are still relatively limited and may not fit the interests of all students. Finally, the vast availability of social media has attracted the attention of college students, resulting in increased time using mobile phones and other electronic devices and reduced participation in in-person and group extracurricular activities.

For many college students, university life is stressful [54]. The stress can be related to financial situations, health, lifestyle, relationships with family, relationships at work/school, problems experienced by loved ones, and more [55]. In this study, 83.23% of the students self-reported no stress, which was different from the results reported by Karyotaki E et al. [55]. The difference may have been related to the use of different survey tools, or based on different cultural contexts and interpretations; stress may be understood as a driving force. In Confucian culture, the internal stress of college students would be transformed into motivation, which promotes positive development. Therefore, stress would not be reported in the survey by those who felt this way. The timing of investigations also differed. This survey was conducted during the COVID-19 pandemic. COVID-19 is an infectious disease to which the population is generally susceptible. It is still a global pandemic; however, the epidemic in China has been brought under control, reducing its contribution to stress among Chinese college students.

This study found a negative correlation between extracurricular activities and stress, which indicated that participating in extracurricular activities led to lower stress, similar to the research results of Kleppang and Fossati [56,57]. Although Spearman’s correlation analysis showed a negative correlation between extracurricular activities and suicidal ideation, mediation analysis showed that extracurricular activities could not directly predict suicidal ideation but could be indirectly predicted through stress. In organizing and participating in extracurricular activities, college students may find various ways to reduce stress and subsequently experience less suicidal ideation. Firstly, extracurricular activities promote social connection, and college students may experience less stress when they are able to rely on emotional support from their peers and teachers. A study by Nir Madjar et al. [58] showed that teacher support and the peer climate have a positive effect on reducing students’ suicidal ideation. In addition, extracurricular activities, especially physical activities, also offer opportunities for college students to express their emotions and release stress.

One study showed that participating in a higher frequency of physical activities and a more regular social life can boost the mental health of college students [59]. In the process of participating in sports activities or social leisure activities, college students often come into contact with many people with shared interests. An increased number of social networks is associated with stronger levels of reciprocal care and more robust social–emotional support. In addition, college students are exposed to new ways of learning and living. A dormitory is an important place for college students to gain support and confidence in these new ways of living and learning, i.e., sharing their emotions and feelings with roommates, establishing emotional closeness with their peers, and reducing their sense of loneliness. Participation in extracurricular activities can facilitate the development of positive interpersonal relationships with others and reduce the social isolation of college students [60]. Reduced loneliness has been shown to have a positive effect on reducing the risk of suicide [61]. Thus, the active participation of college students in extracurricular activities has a positive effect on reducing the psychological stress and suicidal ideation of college students.

The current study demonstrated the positive effect of extracurricular activities on mental health to prevent suicidal ideation among college students. Stress completely mediates the relationship between extracurricular activities and suicidal ideation, and extracurricular activities cannot directly influence suicidal ideation. Based on the current results, it can be hypothesized that the protective effect of extracurricular activities against suicidal ideation is due to a decrease in stress. Participation in extracurricular activities helps college students to experience positive emotions and reduce stress, thus reducing suicidal ideation.

Despite these significant findings, there were numerous limitations in this study. First, it is impossible to infer causality using a cross-sectional study design. Second, suicidal ideation in this study was from the past year. The participants might have had memory bias. In the future, researchers may consider using a longitudinal study design, which has more causal inference ability and will provide a more reliable theoretical basis for the research results.

## 5. Limitations

The study had several limitations. First, the study used cross-sectional data, making it impossible to examine the causal relationship between factors. Our team is building longitudinal cohorts from seven universities, and future studies will fill in this gap. Second, in this study, we analyzed the extracurricular activities as “yes/no”, without reference to which type of extracurricular activities may impact suicidal ideation. Further research in this area is needed in the future.

## 6. Conclusions

In conclusion, the results of this study showed that extracurricular activities have an indirect predictive effect on suicidal ideation in college students. Stress plays a complete mediating role between extracurricular activities and suicidal ideation. Based on these results, there are a number of implications for the holistic education and mental health promotion of college students’ mental health in China. Extracurricular activities that promote social networks, relationship building, and peer support could be integrated into mental health promotion activities on campus. Surveys of student interests could be used to inform the design of extracurricular activities to promote increased participation, especially among students who experience high levels of stress and social isolation. Counselors and teachers could provide structured support around time management to new college students to enable them to take part in extracurricular activities alongside the demands of self-development, relationship building, and academic achievement.

## Figures and Tables

**Table 1:** Demographic comparison of suicidal ideation [mean (SD), n (%)].

Variables	N	Suicidal Ideation			Extracurricular Activities			Stress				
		Yes (n_1_ = 1858)	*χ^2^*/*t*	*p*-Value	Yes (n_2_ = 6020)	*χ^2^*/*t*	*p*-Value	Nothing (n_3_ = 5365)	Mild and Medium (n_4_ = 904)	Severe and above (n_5_ = 177)	*χ^2^*/*F*	*p*-Value
Age (Years)	6446	19.46 ± 1.29	1.264	0.206	19.49 ± 1.28	0.277	0.781	19.47 ± 1.28	19.57 ± 1.34	19.58 ± 1.22	1.724	0.062
Gender (female)	4011	1194 (64.26)	4.613	0.032	3787 (62.91)	18.043	<0.001	3405 (63.47)	512 (56.64)	94 (53.11)	21.789	<0.001
School grades			8.486	0.014		14.959	0.001				18.024	0.001
1	2101	652 (35.09)			1926 (31.99)			1751 (32.64)	294 (32.52)	56 (31.64)		
2	2505	680 (36.60)			2361 (39.22)			2135 (39.79)	310 (34.29)	60 (33.90)		
3 and above	1840	526 (28.31)			1733 (28.79)			1479 (27.57)	300 (33.19)	61 (34.46)		
Living area (city)	2670	855 (46.02)	22.727	<0.001	2503 (41.58)	0.926	0.336	2227 (41.51)	358 (39.60)	85 (48.02)	4.430	0.109
Subject (medical)	1194	401 (21.58)	16.188	<0.001	1091 (18.12)	91.37	0.002	944 (17.60)	194 (21.46)	56 (31.64)	28.399	<0.001
One-child status (yes)	2582	784 (42.20)	4.979	0.027	2404 (39.93)	0.567	0.451	2154 (40.15)	351 (38.83)	77 (43.50)	1.463	0.481
BMI *			0.699	0.873		8.031	0.045				13.418	0.037
Emaciation	1244	363 (19.54)			1176 (19.53)			1050 (19.57)	158 (17.48)	36 (20.34)		
Normal weight	4114	1172 (63.08)			3844 (63.85)			3439 (64.10)	578 (63.94)	97 (54.80)		
Overweight	855	254 (13.67)			790 (13.12)			688 (12.82)	132 (14.60)	35 (19.77)		
Obese	233	69 (3.71)			210 (3.50)			188 (3.51)	36 (3.98)	9 (5.09)		
Family income status			40.959	<0.001		17.482	<0.001				82.620	<0.001
Poor	901	338 (18.19)			818 (13.59)			664 (12.38)	193 (21.35)	44 (24.86)		
Fair	4756	1322 (71.15)			4446 (73.85)			3999 (74.54)	642 (71.02)	115 (64.97)		
Good	789	198 (10.66)			756 (12.56)			702 (13.08)	69 (7.63)	18 (10.17)		

* BMI: Body Mass Index.

**Table 2:** Spearman correlation coefficients between key study variables.

Variables	1	2	3
1. Suicidal ideation	1.000		
2. Extracurricular activities	−0.039 **	1.000	
3. Stress	0.240 ***	−0.083 ***	1.000

** *p* < 0.01. *** *p* < 0.001.

**Table 3:** The mediation effect of stress on the relationship between extracurricular activities and suicidal ideation.

	Effect	Path	Coefficients	95% CI	*p*-Value
SI	Direct effect	Stress-SI (b)	0.997	0.881, 1.113	<0.001
		EA-SI (c)	−0.198	−0.418, 0.023	0.079
	Indirect effect	EA-stress-SI (a∗b)	−0.159	−0.223, −0.099	<0.001
Stress	Direct effect	EA-stress (a)	−0.159	−0.204, −0.114	<0.001

SI: suicidal ideation; EA: extracurricular activities.

## Data Availability

The data are not publicly available due to privacy and research ethical restrictions.
